# Relationship between performances of 10-time-repeated sit-to-stand and maximal walking tests in non-disabled older women

**DOI:** 10.1186/s40101-016-0100-z

**Published:** 2016-06-27

**Authors:** Naoko Yanagawa, Teruichi Shimomitsu, Masashi Kawanishi, Tetsuo Fukunaga, Hiroaki Kanehisa

**Affiliations:** Japan Health Promotion & Fitness Foundation, 2-6-10 Higashishinbashi, Minato-ku, Tokyo, 105-0021 Japan; National Institute of Fitness and Sports in Kanoya, 1 Shiromizu, Kanoya, Kagoshima 891-2393 Japan

**Keywords:** Field test, Sit-to-stand time, Maximal walking time, Power index, Body mass-based exercise, Intervention study

## Abstract

**Aim:**

Sit-to-stand (STS) test is extensively used to assess the functionality of the lower body in elderly people. This study aimed to examine how the score of STS can be associated with that of maximal walking (MW) tests through a cross-sectional as well as longitudinal analysis for non-disabled older women.

**Method:**

Times taken for a 10-time-repeated STS (STS time) and 5-m MW (MW time) were determined before (pre) and after (post) a 3-month body mass-based exercise program in 154 non-disabled women aged 60 to 79 years. In addition to the time scores, STS and MW power indexes (STS-PI and MW-PI, respectively) were calculated using the following equations: STS-PI = (body height − 0.4) × body mass × 10/STS time and MW-PI = body mass × 5/MW time.

**Results:**

At pre- and post-intervention, STS-PI was significantly correlated to MW-PI, with higher correlation coefficients (*r* = 0.545–0.567, *P <* 0.0001) than those between the two time scores (*r* = 0.271–0.309, *P <* 0.001). The intervention significantly improved STS-time (13.6 ± 3.2 s at pre to 9.4 ± 1.8 s at post, *P <* 0.0001), MW time (2.4 ± 0.3 s to 2.2 ± 0.3 s, *P <* 0.0001), STS-PI (46.5 ± 12.5 to 65.7 ± 12.7, *P <* 0.0001), and MW-PI (112.1 ± 20.2 to 124.2 ± 24.4, *P <* 0.0001). There were significant correlations between the changes of STS and MW times (*r* = 0.281, *P <* 0.001) and between those of STS-PI and MW-PI (*r* = 0.366, *P <* 0.0001).

**Conclusion:**

In elderly women, the performance of sit-to-stand task and its training-induced gain are associated with those of the maximal walking task. In addition, the current results indicated that translation of the performance scores of the sit-to-stand and maximal walking tasks to power indexes may be a useful approach for examining the association between the two tasks.

## Background

Sit-to-stand (STS) test is extensively used as a representative test examining the function of the lower body in older people. In fact, STS test scores have been shown to be associated with those of other functional variables [1]. Notably, many studies have reported significant associations between STS performances and walking speed [2–6]. For elderly individuals, it has been shown that, in addition to the muscle strength capability of the lower extremity, the ability of body balance control also becomes a major physical component for performing successfully STS [6–8] and walking [9–11] tasks. This similarity of physical requirements for the two tasks may be a reason for the aforementioned association between their scores.

Walking speed is a critical component of functional independence and quality of life in elderly people [12], and it can be an outcome measure predicting subsequent mobility-related disability or impaired activities of daily living [13–19]. The execution of both STS and walking test is simple and inexpensive, but walking test requires a certain distance for conducting it. STS test needs only a space for placing one chair. Taking this into account together with the aforementioned association between the scores of the two tasks, we can say that STS performance of elderly people is a convenient measure for understanding their levels of functional independence or mobility and for designing optimal exercise programs to improve them. However, the previous findings on the association between the performances of STS and walking tasks have been mainly derived from patients and very old or disabled men and women [2–6]. For non-disabled elderly people, less information about this subject is available from previous studies.

It is known that there is a disparity from linearity in the relationship between muscle strength and some measures of lower extremity performance [20]. Buchner et al. [21] have observed a non-linear relationship between leg strength and walking speed in elderly individuals, in which the corresponding association for weaker persons was significant but that for stronger persons was not. This is also true for the relationship between muscle strength and STS time [22]. Thus, there is a possibility that the aforementioned association between STS and walking scores for very old or disabled men and women would largely depend on the linear relationships between muscle strength and both scores. In other words, it can be said that, for non-disabled individuals, even if STS score is associated with walking performance, the magnitude of the association might not be so strong as those reported for very old or disabled persons because their relationships between muscle strength and both STS and walking performances would be involved to the non-linear region.

In general, STS test score is presented as the time required to repeat a given number of sit-to-stand movements or the number of sit-to-stand movements performed during a given time. In addition to these, some studies have tried to calculate a power index on the basis of STS score and to examine its relationship with force generation capability of the quadriceps femoris [23] and age-related decline [24, 25]. As evidence supporting the validity of the calculated STS power, a biomechanical study reported a strong association with the measured power value [26]. From the findings of Takai et al. [23], STS power index (STS-PI), calculated by using an equation involving leg length, body mass, height of chair, acceleration of gravity, and time taken for a 10-time-repeated STS as independent variables, was strongly associated with the force generation capability of the quadriceps femoris in non-disabled older people. In their results, a significant association was not found in the time required to perform the STS task. Whether or not this can be applied to walking performance is unknown. However, it has been shown that body mass negatively influences the performances of walking [3] as well as STS [7]. It is reasonable to assume that, if the time score of the walking task is the same, the work and power production during the task should be greater for heavier individuals. Taking these aspects into account together with the aforementioned non-linear relationships between muscle strength and both STS and walking scores, therefore, it is hypothesized that, for non-disabled older people, the association between the two tasks will be understood better by using a power index calculated using the parameters of body mass, distance of body displacement, and required time for completing the task.

It is known that elderly women with less knee extensor strength experience a greater difficulty in performing the standing task than younger women [27]. Hortobagyi et al. [28] reported that older adults performed the activities of daily living, involving climbing up and down stairs and rising from a chair, with nearly the maximal torque-generating capabilities of their knee extensors muscles. These findings suggest that, for older adults, a body mass-based exercise program for the lower body, involving STS task, will be an effective modality for improving the force generation capability of the lower body. In fact, some studies have provided evidence supporting this assumption [29, 30], although the magnitude of the gain in knee extensor strength is influenced by the initial level of knee extensor strength per body mass in the practitioners [30]. In these studies, however, STS and walking performances were not examined. While the effects of training on STS and walking performances in older population have been well documented through various exercise interventions [31], it is unknown how the gains in STS and walking performances are associated with each other. If a body mass-based exercise program involving STS is effective to improve not only STS but also walking performances and these gains are significantly associated with each other, it strengthens to assume that the measurement of STS performance can be a conventional outcome measure assessing mobility.

The present study aimed to elucidate the association between STS and maximal walking (MW) performances in non-disabled older women aged from 60 to 79 years. To this end, we employed a cross-sectional and longitudinal analysis design, in which the subjects were involved in a 3-month exercise program, and determined the times taken for a 10-time-repeated STS test (STS time) and 5-m MW test (MW time) before (pre) and after (post) the intervention. In addition to the time scores, we calculated power indexes to represent the performances on STS and MW tests (STS-PI and MW-PI, respectively). As the intervention program, a body mass-based home exercise program involving STS task was chosen because of the proven and substantial effect of this type of training on force generation capability in elderly women [30]. We hypothesized that (1) STS scores are significantly associated with MW scores at both pre- and post-intervention, with a higher correlation coefficient between the power indexes than that between the two time scores and (2) the body mass-based exercise program improves STS and MW scores, and their training-induced gains are significantly associated with each other.

## Materials

### Subjects

One hundred fifty-four Japanese non-disabled elderly women (age, 67.4 ± 4.9 years; height, 152.9 ± 5.0 cm; body mass, 52.6 ± 7.2 kg; body mass index, 22.5 ± 3.1; mean ± SD) voluntarily participated in this study. In accordance with the American College of Sports Medicine guidelines for exercise testing and prescription [32], all subjects were medically screened prior to participation in the experiment. None used a cane or walking aid, and all were functionally independent in daily life. No subject had a history or evidence of lower extremity pain, unstable cardiovascular disease, or other medical condition that would contraindicate performing the STS and MW tasks. All subjects were non-athletes and not involved any specific physical training program (≥30 min/day and ≥2 days/week). This study was approved by the Ethics Committee of the National Institute of Fitness and Sports in Kanoya and was consistent with the institutional ethical requirements for human experimentation in accordance with the Declaration of Helsinki. The subjects were fully informed of the purpose and risks of the experiment and gave their written informed consent.

### Body mass-based home exercise program

The subjects executed a 3-month body mass-based home exercise program. The content of the program adopted was almost the same as reported in previous studies [29, 30]. It was consisted of five exercises: (1) sitting down and standing up from a chair, (2) hip joint extension and flexion, (3) calf raises, (4) side leg raises in a standing position, and (5) trunk flexion and extension in a seated position. The subjects were asked to perform the five exercises successively in a circuit-training style for 3 min/circuit, at a tempo of around 2 s for each exercise. The duration and number of repetitions for performing each exercise were equal, i.e., almost 35 s/exercise and 16 repetitions/exercise, respectively. The subjects performed the exercises once a week at a local gym as an exercise class and on other days at home. The subjects were asked to record the numbers of circuits performed every day. The examiners confirmed the number of circuits performed over that week in the exercise class. During the intervention period, the subjects performed the exercise program 4–7 days a week (6.3 ± 0.8 days/week), completing 1–4 circuits each day (1.6 ± 0.7 circuits/day).

### Measurements

At pre- and post-intervention, the scores of 10-time-repeated STS and 5-m MW tasks were determined. All subjects participated in the pre- and post-measurements.

#### Anthropometry

Height and body mass were measured using standard techniques to the nearest 0.1 cm and 0.1 kg, respectively.

#### Ten-time-repeated STS test

The time (STS time) required to perform a 10-time-repeated STS test on a molded steel chair (0.40-m height and 0.36-m depth) was determined in accordance with the procedure described in a prior study [23]. In the measurements, the subjects were asked to stand up from a sitting position and then to sit down 10 times as fast as possible. The STS time was recorded using a stopwatch to the nearest 10th of a second. Prior to the test, the subjects were asked to perform STS with submaximal effort to familiarize them to the test procedure. After the completion of the warm-up procedure and 1-min rest, the subjects were encouraged to perform the STS with maximal effort. The difference between pre- and post-intervention values for STS time was calculated and referred to as △STS time.

#### Five-meter maximal walking test

The maximal walking test was executed using a procedure similar to that described by Bohannon et al. [4]. Briefly, the subjects were timed using a stopwatch to the nearest 10th of a second as they walked as quickly as possible over a distance between two marks that were 5 m apart. In the measurements, the subjects were provided with a distance of 3 m before and after the 5 m for acceleration and deceleration, respectively. The subjects performed the maximal walking test two times with an interval of 1 min between the trials. The faster time was taken as the individual’s data and referred to as MW time. The difference between pre- and post-intervention values for MW time was calculated and referred to as △MW time.

### Calculation of power index

STS power index (STS-PI) was calculated using the following equation, which is a modification of an equation described by Takai et al. [23]: STS-PI = {(Ht − 0.4) × body mass × 10}/STS time, where 0.4 (m) and Ht (m) represent the height of the chair and body height, respectively. Takai et al. [23] used leg length instead of body height to calculate the STS-PI. The measurement of the leg length is time consuming and has a technical issue because it needs to specify accurately the locations of the greater trochanter of the femur and the malleolus lateralis. Considering these aspects and allowable time for the measurements including STS and MW times, therefore, we used body height instead of leg length to calculate STS-PI. In a prior examination using 556 Japanese women aged 50 to 94 years [24], the corresponding values calculated by the equation used here were highly correlated with those by the equation developed by Takai et al. [23] (*r* = 0.962). MW power index (MW-PI) was calculated using the following equation: MW-PI = (body mass × 5)/MW time, where 5 (m) represents the distance for measuring STS time. The differences between pre- and post-intervention values for STS-PI and MW-PI were calculated and referred to as △STS-PI and △MW-PI, respectively.

### Statistics

Descriptive values are presented as means ± SDs. A simple linear regression analysis was used to calculate the coefficients of correlations between time scores, between power indexes, and between training-induced changes. An analysis of covariance (ANCOVA) was used to test the differences between regression equations for the relationships between STS time and MW time and between STS-PI and MW-PI at pre- and post-intervention. A Student’s paired *t* test was used to examine the differences between pre- and post-intervention values of each of STS time, MW time, STS-PI, and MW-PI. In addition, effect size (Cohen’s *d*) was calculated to express the magnitude of the difference between the two means of pre- and post-intervention values. The probability level for statistical significance was set at *P <* 0.05.

## Results

No one dropped out from the intervention program. Table 1 summarizes the descriptive data on the measured and calculated values for pre- and post-intervention. There were no significant changes in body height, body mass, and body mass index. The intervention significantly improved the time scores of STS and MW and increased the power indexes of the two tasks. Cohen’s *d* values in time score and power index were greater in STS than in MW (Table 1).

**Table 1:** Descriptive data at pre- and post-intervention

Variables	Pre-intervention	Post-intervention	△	Cohen’s *d*
Body height, cm	152.6 ± 4.4	152.6 ± 4.4	0.0 ± 0.5	0.00
Body mass, kg	53.0 ± 6.3	53.1 ± 6.3	0.1 ± 0.9	0.11
STS time, s	13.6 ± 3.2	9.4 ± 1.8^*^	−4.2 ± 2.8	−1.50
MW time, s	2.4 ± 0.3	2.2 ± 0.3^*^	−0.2 ± 0.3	−0.67
STS-PI	46.5 ± 12.5	65.7 ± 12.7^*^	19.2 ± 11.5	1.67
MW-PI	112.1 ± 20.2	124.2 ± 24.4^*^	12.1 ± 18.0	0.67

*STS time* time required to perform 10-times-repeated sit-to-stand task, *MW time* time required to perform 5-m maximal walking task, *STS-PI* sit-to-stand power index, *MW-PI* maximal walking power index

^*^Significantly different from the pre-intervention value at *P <* 0.0001

**Fig. 1:**
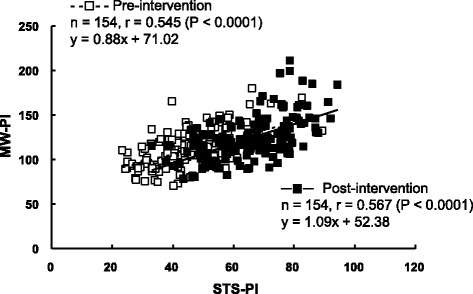
Relationships between STS-PI and MW-PI at pre- and post-intervention

STS time was significantly correlated with MW time at pre- (*r* = 0.309, *P <* 0.001) and post-intervention (*r* = 0.271, *P <* 0.001). STS-PI was also significantly correlated to MW-PI, with higher correlation coefficients than those between the time scores: *r* = 0.545 (*P <* 0.0001) at pre-intervention and *r* = 0.567 (*P <* 0.0001) at post-intervention (Fig. 1). In this relationship, while there was no significant difference between the slopes of the regression lines for the relationships between STS-PI and MW-PI at pre- and post-intervention (*F* = 1.517, *P =* 0.219), the *y* intercept was significantly different between the two regression lines (*F* = 6.741, *P <* 0.01).

**Fig. 2:**
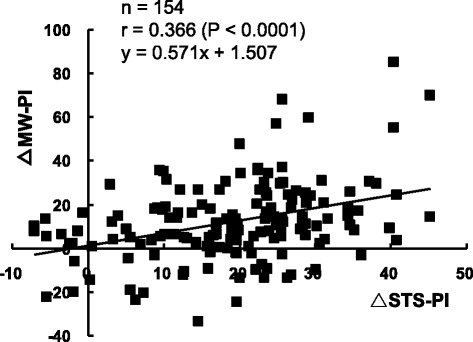
Relationship between the changes of STS-PI and MW-PI at post-intervention

△STS time was significantly correlated to △MW time (*r* = 0.281, *P <* 0.001). Similarly, △STS-PI was significantly correlated to △MW-PI (*r* = 0.366, *P <* 0.0001, Fig. 2).

## Discussion

At pre- and post-intervention, there were significant associations between the time scores and between the power indexes of STS and MW. The directions of body movement while executing STS and MW are different: vertical movement in STS and horizontal movement in MW. As described earlier, however, both STS and walking performances can be associated with common physical components, i.e., muscle strength and balance. In addition, Brach et al. [33] reported that elderly women with a higher slope of the electromyogram activities of the quadriceps femoris during STS walked faster. Furthermore, DeVita and Hortobagyi [34] observed that elderly individuals used their hip extensors more than young adults when walking at the same speed. It is known that, for older men and women, the performances of both STS and MW are strongly associated with the power production during leg extension movement which involves extension of the knee and hip joints [5]. These findings indicate that, for elderly people, the function and use of the lower body muscles, being required to achieve better performances in STS and MW, are similar between the two tasks. At the same time, this will be a reason why the performances of STS and MW were significantly associated with each other.

Many studies have already reported significant associations between the time scores of STS and walking for relatively short distances [2–6]. In these studies, the reported correlation coefficients ranged from 0.48 to 0.71. Compared to these values, our observed correlation coefficients between STS and MW times (*r* = 0.303 at pre-intervention and *r* = 0.267 at post-intervention) are lower. However, most of the previous findings cited above have been obtained by examining patients [3, 4] and very old [5, 6] or disabled [2] men and women. The current study examined elderly women who were functionally independent in daily life and completed the two tasks without support and large difficulty. In the report of Seeman et al. [35], who examined a high-functioning elderly cohort, the correlation coefficient between the time scores of 10-ft maximal walking and 5-time-repeated STS was 0.21. Thus, the previous and current studies indicate that the association between STS and MW times for non-disabled elderly individuals is not so strong as compared to that reported previously for very old or disabled men and women.

Physiological reasons for the observed lower correlation coefficient between STS and MW times are unknown but might involve the possible difference between disabled and non-disabled individuals in the relationships between muscle strength and the two time scores. As described earlier, the time scores of rising from a chair and walking over a certain distance in older adults have been shown to be related to the force generation capability of the lower limb muscles in a curvilinear fashion, in which a more linear relationship exists in weaker individuals [20, 22, 26, 36]. Ferrucci et al. [20] reported that, in the lower range of the spectrum of strength, various measures of lower extremity performances were associated with strength, while the relationship above a certain threshold of strength was limited to only some of these performances. On the basis of electromyogram recordings and calculation of knee joint moments, Hortobagyi et al. [28] have reported that old adults perform activities of daily living with near their maximal capabilities. From the findings of Fujita et al. [37], the activation level of knee extensor muscles during body mass-based squat movement was significantly higher in frail elderly persons, who used the long-term care insurance system, than in older individuals who were functionally independent. Considering these findings, it seems that the similarity between STS and MW tasks in physical requirements for performing each of the two tasks, notably in muscular effort, would be weak in non-disabled than in disabled persons, and consequently, it might have resulted in the lower correlation coefficient between STS and MW times in the non-disabled individuals.

As hypothesized at the start of this study, the correlation coefficient between STS-PI and MW-PI (*r* = 0.547 at pre-intervention and *r* = 0.560 at post-intervention) was higher than those between STS and MW times. In both STS and MW tasks, the body mass of the examinee becomes a load for the exercising muscles of the lower body. As described earlier, some studies have indicated that body mass negatively influences the performances of either STS [7] or maximal walking [3]. For example, Lord et al. [7] reported that, in addition to muscle strength relative to body mass, body mass was an independent negative variable in a prediction equation for five-time-repeated STS time in community-dwelling older men and women. For maximal walking performance, Bohannon et al. [3] indicated that, as a result of multiple regression analysis, body mass was a negative independent variable for predicting the fastest walking speed in kidney transplantation candidates. These findings suggest that, when one tries to examine the association between the performances of STS and MW tasks, the influence of body mass cannot be ignored. In this sense, the translation of time scores to power index is a better approach for examining the association between the performance scores of STS and MW. The current results support this idea.

In previous studies, an inconsistency has been found between the individual differences observed in muscle function and the performance scores of mobility. For example, the sex difference in STS time or walking speed does not always reflect that in knee extensor strength [23, 38] or leg extension power [5]. Takai et al. [23] reported that, for non-disabled older people, the STS power index was strongly associated with the force generation capability of the knee extensors, but the time required to perform the STS task was not. Furthermore, Feland et al. [39], who examined sit-to-stand performance in senior athletes aged 50 years and older, reported that rising power had significant effects of age and sex, but weight transfer time remained similar regardless of age or sex. Considering these findings together with the current results, we may say that the translation of performance scores to power scores can provide a consistency in the assessment of the relationships among different performance scores and their associations with muscle function such as strength and power.

STS and MW performances significantly increased after the intervention. Many studies have already reported positive effects of exercise interventions on the performances of STS and/or MW tasks in frail elderly people [31]. While the exercise modalities adopted in previous studies vary among reports [31], most of these studies have adopted exercises with an external load or elastic band besides body mass-based exercises. The training program used here consisted of only body mass-based exercises involving STS task. This training style has been shown to improve the force generation capability of the quadriceps femoris in elderly women [30] and men [29]. However, no information on the effectiveness of the body mass-based exercise program for STS and MW performances is available from the two studies. The current study provides evidence on this point.

In addition to the significant gains in time scores and power indices of STS and MW tasks, their changes were significantly associated with each other. These results support our hypothesis and strengthen to assume that STS test score can be a convenient and representative measure for assessing mobility in older women. As described earlier, not only muscle strength but also balance can be a major physical component for elderly people to perform successfully STS and walking [6–11]. The present study did not determine muscle strength and balance, and so it is unknown how the intervention adopted here changed these variables and their changes can be associated with those in STS and MW scores. However, some studies have reported that, for frail elderly people, exercise-induced gains in lower extremity strength are associated with those in STS and/or walking performances [12, 40, 41]. It has been shown that the training program used here can improve the force generation capability of the quadriceps femoris [29, 30]. In addition, it involved tasks exercising muscles around the hip and ankle joints. Thus, as a physiological background for the observed association between the gains in the scores of STS and MW tasks, the possible gains in the force generation capability of lower extremity muscles might be considered. In the current results, however, it should be noted that the *y* intercept of the regression line for the relationship between STS-PI and MW-PI was significantly different between pre- and post-intervention, although no corresponding difference was found in the slope. As shown in Table 1, the Cohen’s *d* values indicated that the magnitude of the difference between the two means of pre- and post-intervention values was greater in STS-PI than in MW-PI. This may be due to that STS was involved as an exercise task, i.e., the influence of task-specificity, and at the same time, it would be a reason for the observed significant difference in the *y* intercept. In addition, it should be remarked that there is evidence suggesting that the training-induced change in STS performance is not always associated with that in walking performance. For example, Schlicht et al. [42] reported that, in comparison with a control group, intense resistance training for older adults produced a significant gain in maximal walking speed without the corresponding change in STS performance. Conversely, the findings of Cao et al. [43] indicated that a combined exercise intervention program significantly increased STS score but did not increase maximal walking performance. Thus, we cannot rule out that the observed association between the training-induced gains of STS and MW scores might have reflected the content of the exercise program adopted here. With regard to the association between the training-induced gains of the two variables, further study is needed to clarify the influence of the content of intervention program.

## Conclusion

The current results indicate that, in non-disabled older women, the performance of the sit-to-stand task and its training-induced gain are associated with those in the maximal walking task. The translation of the performance scores of the sit-to-stand and maximal waking tasks to power indexes may be a useful approach for examining the association between the two tasks.

## Abbreviations

ANCOVA, analysis of covariance; MW test, maximal walking test; MW time, Time for a 5-m maximal walking test; MW-PI, power index calculated from the score of a 5-m maximal walking test; STS test, sit-to-stand test; STS time, time for a 10-times-repeated STS test; STS-PI, power index calculated from the score of a 10-times-repeated STS test; △MW time, difference between the MW times at pre- and post-intervention; △STS time, difference between the STS times at pre- and post-intervention; △MW-PI, difference between the MW-PI values at pre- and post-intervention; △STS-PI, difference between the STS-PI values at pre- and post-intervention
